# Structure and function of cytidine monophosphate kinase from *Yersinia pseudotuberculosis*, essential for virulence but not for survival

**DOI:** 10.1098/rsob.120142

**Published:** 2012-12

**Authors:** Nicola J. Walker, Elizabeth A. Clark, Donna C. Ford, Helen L. Bullifent, Erin V. McAlister, Melanie L. Duffield, K. Ravi Acharya, Petra C. F. Oyston

**Affiliations:** 1 Biomedical Sciences, Defence Science and Technology Laboratory, Porton Down, Salisbury SP4 0JQ, UK; 2 Department of Biology and Biochemistry, University of Bath, Claverton Down, Bath BA2 7AY, UK

**Keywords:** cytidine monophosphate kinase, crystal structure, virulence, *Yersinia*

## Abstract

The need for new antibiotics has become pressing in light of the emergence of antibiotic-resistant strains of human pathogens. *Yersinia pestis*, the causative agent of plague, is a public health threat and also an agent of concern in biodefence. It is a recently emerged clonal derivative of the enteric pathogen *Yersinia pseudotuberculosis.* Previously, we developed a bioinformatic approach to identify proteins that may be suitable targets for antimicrobial therapy and in particular for the treatment of plague. One such target was cytidine monophosphate (CMP) kinase, which is an essential gene in some organisms. Previously, we had thought CMP kinase was essential for *Y. pseudotuberculosis*, but by modification of the mutagenesis approach, we report here the production and characterization of a *Δcmk* mutant. The isogenic mutant had a growth defect relative to the parental strain, and was highly attenuated in mice. We have also elucidated the structure of the CMP kinase to 2.32 Å, and identified three key residues in the active site that are essential for activity of the enzyme. These findings will have implications for the development of novel CMP kinase inhibitors for therapeutic use.

## 2. Introduction

Cytidine monophosphate (CMP) kinase is a member of the nucleoside monophosphate (NMP) kinase family, and plays a crucial role in biosynthesis of nucleoside precursors. In bacteria, CMP kinase catalyses the transfer of a phosphoryl group from ATP to CMP or dCMP. This differs from the activity of eukaryotic CMP kinases, which catalyse the conversion of UMP and CMP to the respective diphosphate form [1], and, structurally, bacterial CMP kinases are different from other members of the NMP kinase family [2]. CMP kinase over-expression can suppress the lethal effects of inactivation of the UMP kinase, PyrH [3,4], which indicated a low level of UMP kinase activity in CMP kinase, an observation confirmed later [5].

CMP kinase has been reported to be essential for viability of *Bacillus subtilis* [6] and *Streptococcus pneumoniae* [7]. However, inactivation of *cmk* is reportedly not lethal in *Escherichia coli* [8] nor in *Salmonella enterica* [9]. In *Haemophilus influenzae*, there are two copies of *cmk* [10], but this has not been reported for *E. coli*. However, there are conflicting data regarding whether *cmk* is essential in *E. coli*, and other mutagenesis studies indicate that *cmk* is, in fact, essential in this organism [11,12], so essentiality of this target may be influenced by strain or method used. Where a mutant was generated, the *E. coli* mutant had a significant growth defect and demonstrated cold sensitivity [8]. Previously, we had attempted to create a *cmk* mutant in *Yersinia pseudotuberculosis*, without success, which indicated that *cmk* is an essential gene in this member of the Enterobacteriaceae [13].

*Yersinia pseudotuberculosis* is one of three human pathogenic members of the genus, the others being *Yersinia enterocolitica* and *Yersinia pestis*. Although *Y. pseudotuberculosis* and *Y. enterocolitica* are enteric pathogens, *Y. pestis* causes bubonic and pneumonic plague. *Yersinia pestis* is considered to be a recently emerged clonal derivative of *Y. pseudotuberculosis* [14]. The evolution of *Y. pestis* from enteropathogen to an arthropod-vectored systemic pathogen has involved both gene acquisition and loss [15], yet the two organisms retain high levels of genetic similarity. As a result, *Y. pseudotuberculosis* is often exploited as a safer model pathogen for elucidating pathogenic mechanisms of *Yersinia*, as proteins often retain their function in both species, and results can then be validated subsequently in the plague bacillus in more focused efforts.

Approximately 2000 cases of plague are reported annually to the World Health Organization. In the case of suspected plague infection, the recommended antibiotics for therapy are gentamicin and doxycycline or quinolones [16], with prompt initiation of therapeutic regimens essential, particularly for the highly acute pneumonic form of disease. Antibiotic resistance has been reported in clinical isolates [17], as has highly efficient transfer of resistance genes in the midgut of the flea [18]. As human-to-human transmission is a serious risk in plague cases, there is a need for effective vaccines and antibiotics to prevent outbreaks arising from enzootic areas.

Previously, we have reported a bioinformatic approach to identify potential new targets for novel antimicrobials [13]. The database of essential genes (DEG) records those genes thought to be essential in a range of bacteria. Using a down-selection process that included conservation within DEG, *cmk* was identified as a potential lethal gene, with *cmk* homologues identified in 11 of the 14 prokaryotes listed in DEG. Other selection criteria, including size, enzymatic function and non-membrane protein, provided additional support for CMP kinase as a ‘druggable’ target through inhibition of its active site. In this study, we wished to definitively prove whether CMP kinase was an essential locus when mutated. We have elucidated the crystal structure of CMP kinase from *Y. pseudotuberculosis* and validated the function of the enzyme as a kinase. Through homology modelling, we have identified potentially important protein–ligand interactions and elucidated the essentiality of a number of these interactions by site-directed mutagenesis. These results thus provide the basis for further research into the development of novel therapeutics for plague, and for inhibitors of bacterial CMP kinase that may represent a novel class of broad-spectrum antimicrobials.

## 3. Material and methods

Unless otherwise stated, chemicals were purchased from Sigma-Aldrich (Poole, UK), and enzymes were purchased from Promega Ltd (Southampton, UK).

### 3.1. Production of *Yersinia pseudotuberculosis Δcmk* mutant

Construction of the *Y. pseudotuberculosis Δcmk* mutant was generated as reported previously [13] with the modification that the primers were designed to amplify the kanamycin-resistance cassette without including the cognate promoter region. The primers used were: 5′-CTGCCGGGGGCAGACAAGAGATTTGCCTACCGAAAGGAGAGATAA*ATGAGCCATATTCAACGGG*-3′ (forward) and 5′-CGTATTGAGAGCGCAGAACTGACCGCATTGTCG*TTAGAAAAACTCATCGAGCATC*-3′ (reverse), where the sequence in italic font hybridized to the kanamycin-resistance gene. PCR products were generated using the plasmid pK2 [19] as a template, and excess template was digested with *Dpn*I. PCR products were purified using Millipore Microcon Ultracel YM-100 and were then transformed into *Y. pseudotuberculosis* IP32953 [20] pAJD434 [21] by electroporation. Following overnight incubation at 28°C in Luria Bertani (LB) broth supplemented with 0.8 per cent arabinose, transformants were selected on LB agar supplemented with kanamycin (50 μg ml^−1^) and trimethoprim (100 μg ml^−1^) for 48 h at 28°C. Transformants were screened by PCR using target gene-specific (5′-TTTGCCTACCGAAAGGAGAG-3′ and 5′-GCGCAGAACTGACCGCATTG-3′) and kanamycin gene-specific primers (5′-GCCATATTCAACGGGAAACG-3′ and 5′-AAACTCACCGAGGCAGTTCC-3′).

Mutant strains were cured of the pAJD434 plasmid by growth at 37°C in LB medium supplemented with kanamycin (50 μg ml^−1^). Cured mutant strains were screened for the virulence plasmid pYV by PCR for two genes located on this plasmid: *virF* and *yscC*. The retention of the *Yersinia* virulence plasmid (pYV) was also confirmed by culture on Congo-red magnesium oxalate plates, where plasmid retention results in small red colonies and plasmid loss results in large pink colonies.

Mutation of *cmk* was confirmed by Southern blotting. Genomic DNA was digested overnight with *Bpu*10I, resolved on a 0.7 per cent agarose gel and blotted onto a positively charged nylon membrane (Roche). Southern hybridization was performed using a digoxigenin-labelled probe amplified from the open reading frame located immediately downstream of *cmk* encoding the 30S ribosomal protein S1 (*rpsA*; primer sequences 5′-GAAAGCTAAGCGTCACGAAG-3′ and 5′-GTCCAAGCCGATGAAGATAC-3′) and the Roche DIG system, according to the manufacturer's instructions.

### 3.2. Expression and purification of CMP kinase

The *Y. pseudotuberculosis cmk* gene was amplified by PCR from genomic DNA using Phusion DNA polymerase (Finnzymes, NEB) and the oligonucleotide primers GSTcmkfor 5′GGATCCATGACGGCGATAGCCCCGGTGATA3′ (*Bam*H1 site indicated) and GSTcmkrev 5′GCGGCCGCTTATTTTTTCAACGGCAAGG3′ (*Not*1 site indicated). The amplified fragment was ligated into the blunt cloning vector pCR-Blunt II-TOPO (Invitrogen) using T4 DNA ligase (Roche). The *cmk* insert was excised from the plasmid by restriction digestion with *Bam*H1 and *Not*1, and ligated into the pGEX-6P-1 (GE Healthcare) vector. Fidelity of the *cmk* gene was confirmed by sequencing.

*Escherichia coli* BL21*(DE3) harbouring recombinant pGEX-6P-1 plasmids were cultured in LB broth supplemented with 1 per cent glucose and ampicillin (50 μg ml^−1^). Cultures were grown with shaking (170 rpm) at 37°C until an optical density at 600 nm (OD_600_) of 0.4 was reached. Protein expression was induced with 1 mM isopropyl-B-d-thiogalactoside (Roche) with incubation for a further 4 h followed by harvesting by centrifugation (10 min, 1700 g). Cell pellets were resuspended in PBS and were sonicated in an ice water bath. The suspension was clarified by centrifugation at 27 000*g* for 30 min. Supernatants were loaded onto a GSTrap FF column (GE Healthcare) equilibrated with PBS. The column was washed with equilibration buffer, followed by cleavage buffer (50 mM Tris, 150 mM NaCl, 1 mM EDTA, 1 mM DTT pH 7). PreScission Protease (GE Healthcare) in cleavage buffer was added (160 units per millilitre of column matrix) and incubated overnight at 4°C. The column was then washed with cleavage buffer, and fractions containing cleaved CMP kinase were pooled and buffer exchanged into 50 mM HEPES, 150 mM NaCl, 10 mM glycerol pH 7.

For structural analysis, elution fractions containing CMP kinase as determined by SDS–PAGE analysis were pooled and buffer exchanged into 50 mM MES pH 6.0, 150 mM NaCl. Protein was concentrated to approximately 6.4 mg ml^−1^, using a Millipore concentrator.

### 3.3. Crystallization of CMP kinase

Initial screens to identify conditions in which CMP kinase crystallized were carried out using pre-prepared 96-well screens (Molecular Dimensions) on an Art Robbins Phoenix nano-dispenser. Drops of 300 nl were set up using the sitting drop method. Optimization was carried out in 24-well plates using the hanging drop method. Improved crystals of CMP kinase grew over 7 days in a 2 μl drop that contained a 1 : 1 volume ratio of protein solution to mother liquor. Mother liquor contained 100 mM sodium HEPES pH 7.5, 1.2 M lithium sulphate and 100 mM magnesium chloride.

### 3.4. Structure determination and refinement

X-ray diffraction data were collected from a single cryo-cooled crystal (100 K) with 2 M lithium sulphate at the Diamond Light Source (Didcot, UK) on an ADSC Q315 CCD detector on station I02 (*λ* = 0.98 Å). Diffraction images were collected at an oscillation angle of 1.0°. Data were processed in the rhombohedral space group R3 using XDS [22], and data were scaled using SCALA [23]. Initial phases were obtained through molecular replacement using the program Phaser [24]. The search model used for molecular replacement was a model of *Yersinia* CMP kinase generated based on its sequence alignment with *E. coli* CMP kinase (PDB code 1CKE). Model building was performed with COOT [25], and refinement was performed with PHENIX [26]. A set of reflections (5%) was set aside for *R*_free_ calculation [27].

### 3.5. Homology modelling

A model of *Yersinia* CMP kinase was created using the SWISS modeller server [28]. This model was based on the sequence alignment between *Yersinia* CMP kinase and *E. coli* CMP kinase with CDP bound (PDB code 2CMK). The coordinates from 2CMK were used to add CDP to the model.

### 3.6. CMP kinase activity assay

CMP kinase activity was determined using a coupled spectrophotometric assay based on that of Blondin *et al.* [29]. Briefly, for the determination of the *K*_m_ for CMP, 1 ml of 50 mM Tris pH 7.4 containing 2 mM MgCl_2_, 50 mM KCl, 1 mM phosphoenolpyruvate, 0.2 mM NADH, 1 mM ATP, two units each of lactate dehydrogenase, pyruvate kinase and NDP kinase, and various concentrations of CMP, or for the determination of the *K*_m_ for ATP 0.1 mM CMP and various concentrations of ATP was incubated at 30°C and the absorbance monitored at 340 nm. When a stable absorbance was reached (10 µM), CMP kinase was added and the decrease in absorbance recorded.

### 3.7. Generation of point mutations in CMP kinase

Point mutants Arg188Ala, Arg131Ala and Arg110Ala were generated using the Stratagene Quikchange site-directed mutagenesis kit. Mutagenesis was carried out within the recombinant *cmk*-pGEX-6P-1 plasmids, using oligonucleotide primers 5′-GGATAACCGTGATCGTAACGCTGCTGTTGCACCTTTAGTC^-^3′, 5′-GGTTTAATTGCTGATGGCGCGGATATGGGGACTATCGT-3′ or 5′-CGGCTTTCCCCCGAGTAGCTGAAGCATTACTGC^-^3′. Mutated plasmids were transformed into *E. coli* XL-1 blue and plated onto LB plates supplemented with ampicillin (50 µg ml^−1^). Colonies were screened by PCR and sequenced using the T7 universal primers.

### 3.8. Circular dichroism spectroscopy

Circular dichroism (CD) studies were performed on a Jasco J-600 spectropolarimeter with a 2 mm circular cell. Samples of wild-type CMP kinase (0.7 mg ml^−1^) and each of the mutants Arg188Ala (0.3 mg ml^−1^), Arg131Ala (0.4 mg ml^−1^) and Arg110Ala (0.7 mg ml^−1^) were prepared in 50 mM HEPES, 150 mM NaCl and 10 mM glycerol pH 7.0. Wavelength scans were performed at 20°C from 195 to 250 nm at a rate of 50 nm min^−1^.

### 3.9. *In vitro* evaluation of the growth of the *Δcmk* mutant

At 28°C with agitation, cultures of strain IP32953 and the *Δcmk* mutant were grown overnight in LB or LB supplemented with 50 μg ml^−1^ kanamycin, respectively. The overnight cultures were used to inoculate fresh pre-warmed medium, and the cultures were incubated as above. At selected intervals, aliquots were removed for OD_600_ measurement and enumeration of viable counts.

*In vitro* competition index (CI) experiments were conducted prior to *in vivo* CI evaluation. Cultures of both strains were grown in LB overnight at 28°C with agitation, and diluted to an OD_600_ of 0.1 in LB. These cultures were serially diluted and viable counts determined by culture on LB agar. Wild-type and mutant bacterial suspensions were mixed in a 1 : 1 ratio and incubated at 28°C with shaking. At selected intervals, aliquots were removed for OD_600_ measurement and enumeration of viable counts. The CI is defined as the output ratio (mutant/wild-type) divided by the input ratio (mutant/wild-type).

### 3.10. *In vivo* studies with the *Δcmk* mutant

Attenuation of the *Y. pseudotuberculosis Δcmk* mutant was evaluated by CI and median lethal dose (MLD) evaluation. Mutant and wild-type strains were grown separately in 100 ml LB broth with shaking at 28°C overnight. The cultures were then centrifuged (10 min, 6000*g*) and each pellet resuspended in 4 ml sterile 10 per cent v/v aq. glycerol. Aliquots were stored at −80°C until required. One aliquot of each strain was defrosted, serially diluted and cultured to determine cfu ml^−1^.

For the CI challenge, one aliquot of each strain was defrosted, diluted in PBS to approximately 1 × 10^9^ cfu ml^−1^, and wild-type and mutant bacterial suspensions mixed in a 1 : 1 ratio. This suspension was then serially diluted with sterile PBS. Retrospective viable counts were determined by plating out dilutions in triplicate on LB agar and LB agar supplemented with kanamycin to determine the input ratio. Groups of four mice were then dosed with 0.1 ml of these dilutions by the intravenous route. After 3 days, spleens were removed and passed through 70 μm sieves (Becton–Dickinson) to produce a cell suspension in 3 ml of PBS. Cell suspensions were serially diluted in sterile PBS and plated onto LB agar, and LB agar supplemented with kanamycin to determine the output ratio. The CI was determined as above.

For MLD determination, an aliquot of each culture was defrosted and diluted in PBS. Retrospective viable counts were determined by plating out dilutions in triplicate on LB agar. Groups of five mice were dosed intraperitoneally (i.p.) with 0.1 ml of these dilutions. Mice were observed twice daily, and animals that were moribund and deemed incapable of survival were humanely killed. Mice were observed for 28 days. The MLD was calculated as reported [30].

## 4. Results

### 4.1. Inactivation of CMP kinase is highly attenuating

The *Y. pseudotuberculosis cmk* gene was inactivated by replacement of the gene with a kanamycin-resistance cassette driven by the *cmk* promoter. Following transformation with the PCR product, three colonies grew on LB kanamycin agar, and were selected for screening by PCR. Of these, all were shown to be mutants lacking the *cmk* gene but carrying the kanamycin cassette. Following curing of the pAJD434 plasmid, and confirmation that the unstable *Yersinia* virulence plasmid pYV had been retained, the mutation in the three mutants was confirmed by Southern blotting, where a size difference of approximately 4 kb was observed between the *Δcmk* mutant and wild-type.

**Figure 1.**
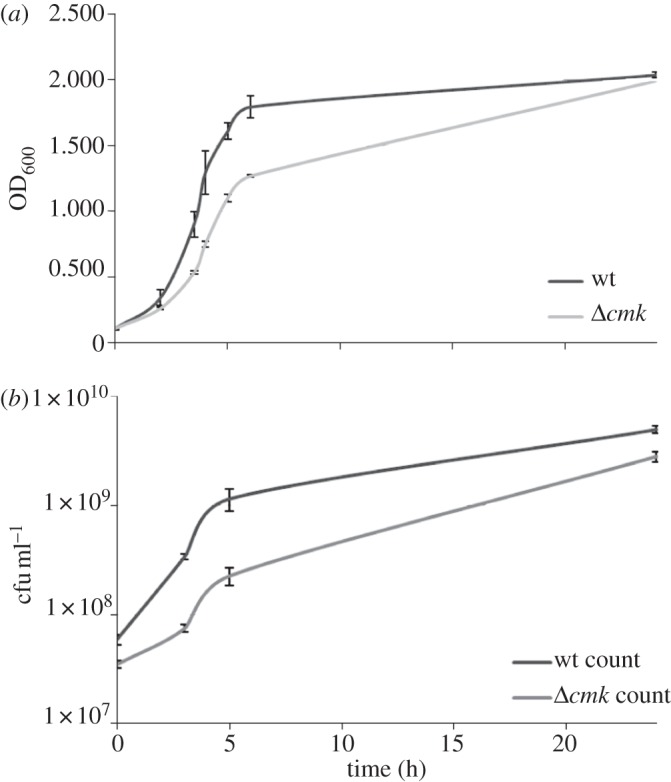
Growth of *Y. pseudotuberculosis* IP32953 and the *Δcmk* mutant, followed by (*a*) optical density and (*b*) viable count.

In pure culture, the growth of the *Δcmk* mutant appeared slower than that of the wild-type, although it eventually reached a similar final density (figure 1). A fitness defect was also observed by *in vitro* CI, where a value of 0.186 indicated reduced fitness of the mutant compared with wild-type.

**Figure 2.**
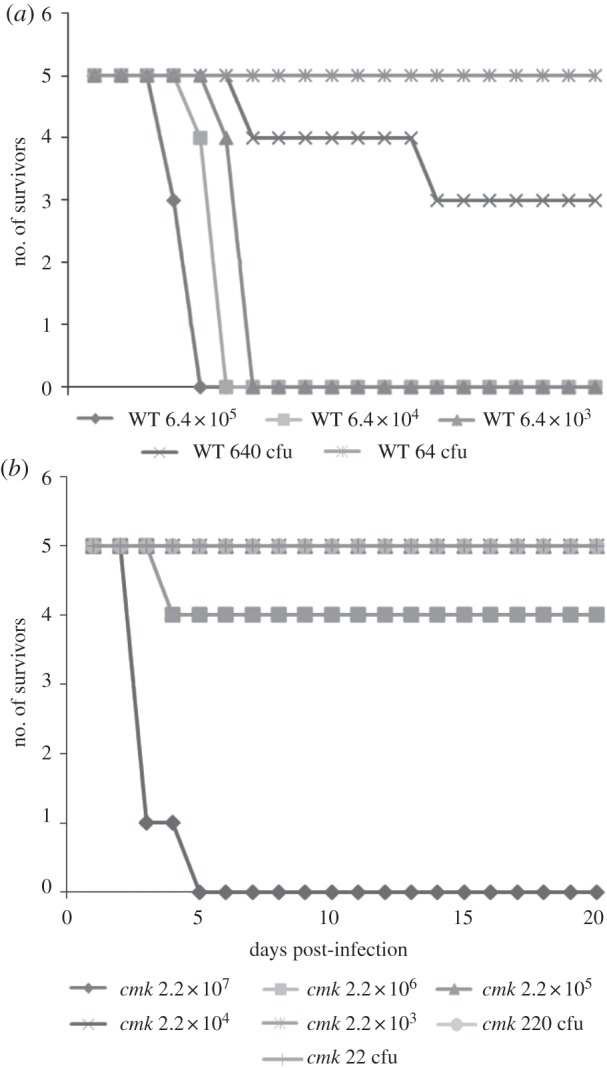
Survival of mice following challenge with (*a*) wild-type *Y. pseudotuberculosis* IP32953 and (*b*) the *Δcmk* mutant.

Mice were challenged i.p. with serial dilutions of wild-type *Y. pseudotuberculosis* and the *Δcmk* mutant. Retrospective challenge doses showed that mice challenged with IP32953 received 6.4 × 10^5^–0.64 cfu, while mice challenged with the mutant received 2.2 × 10^7^–22 cfu. The published MLD of the wild-type strain is 2200 cfu [31]. In this study, the MLD of the wild-type strain was 940 cfu. The *Δcmk* mutant was significantly attenuated, with an MLD of 9.28 × 10^7^ cfu (figure 2), and no signs were recorded for mice receiving 2.2 × 10^5^ cfu or less of the mutant. This was confirmed by *in vivo* CI, where a CI of 4.48 × 10^−6^ indicated a significant reduction in fitness compared with the wild-type (a CI of 0.2 or below is considered a significant reduction in fitness in this model).

### 4.2. The CMP kinase crystal structure

There is one CMP kinase molecule in the crystallographic asymmetric unit, and the solvent content is 48 per cent. The final refined structure has an *R*_cryst_ of 22.2 per cent and an *R*_free_ of 27.8 per cent (table 1). The final model consists of 18 water molecules and two sulphate ions in the asymmetric unit. The Ramachandran plot generated by Molprobity shows that three residues are in disallowed regions [32]. Clear density is observed from Met1 through Ala225 with the exception of a 12-residue gap from Glu180 to Ala191. The five N-terminal residues are not observed. Side chain density is not observed for residues Glu57, Glu58, Arg92 and Glu122, and so these side chains have been modelled.

**Table 1.** Crystallographic data for the CMP kinase structure. Numbers in parentheses are for the upper resolution shell (2.32–2.45 Å), where appropriate.

cell dimensions	*a* = *b* = 88.64 Å , *c* = 84.29 Å; *α* = *β* = 90°, *γ* = 120°
space group	R3
resolution (Å)	2.32
completeness (%)	96.4 (87.4)
no. of reflections	44 472(4160)
no. of unique reflections	10 266(1370)
redundancy (%)	4.3 (3.0)
*I*/σ*I*	16.2 (2.8)
*R*_merge_ (%)^a^	4.7 (46.4)
*R*_cryst_ (%)^b^	22.2
*R*_free_ (%)^c^	27.8
Wilson B-factor (Å^2^)	61.6
average B-factors main chain/side chain (Å^2^)	64.2/67.2
r.m.s.d. from ideal values	
bonds (Å)	0.008
angles (°)	1.168

^a^*R*_merge_ = *Σ_hkl_*Σ*_i_*|*I_i_*(*hkl* − 〈*I*(*hkl*)〉|/*Σ_hkl_*Σ*I_i_*(*hkl*), where 〈*I*〉 is the averaged intensity of the *i* observations of reflection *hkl*.

^b^*R*_cryst_ = *Σ*||*F*_o_|−|*F*_c_||/*Σ|F*_o_*|*, where *F*_o_ and *F*_c_ are observed and calculated structure factors, respectively.

^c^*R*_free_ is equal to *R*_cryst_ for a random set of reflections (5%) not used in refinement [27].

**Figure 3.**
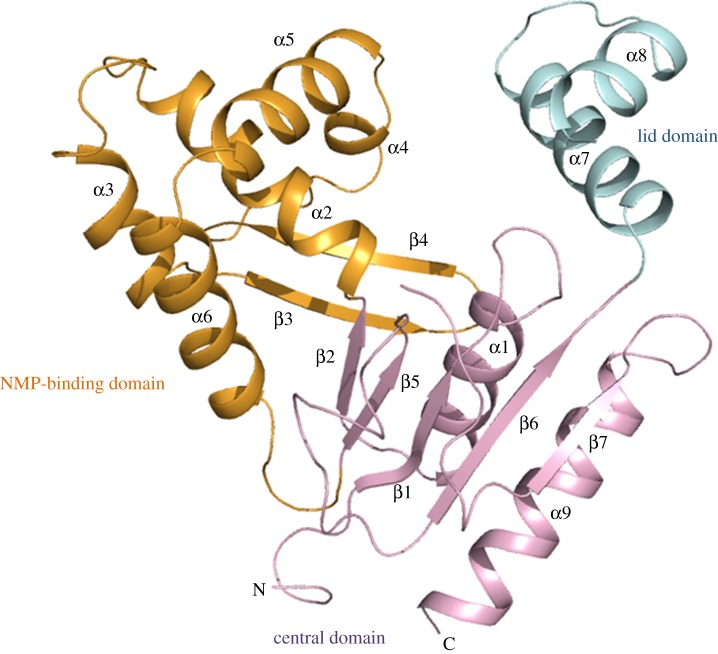
The overall fold of CMP kinase is shown. α-helices and β-strands are numbered. The N- and C-termini are labelled. The central domain, lid domain and NMP-binding domains are coloured pink, blue and orange, respectively. This figure was prepared using PyMOL (v. 0.99; Schrödinger, LLC; www.pymol.org).

*Yersinia* CMP kinase adopts the same overall fold as related CMP kinases and is composed of nine α-helices and seven β-strands connected by loops. These are arranged into three domains (figure 3). The central portion of the molecule is composed of a central β-sheet, made up from β1, β2, β5, β6 and β7, and α1 and α9. Helix α6 joins the central domain to the NMP-binding domain. The NMP-binding domain is composed of anti-parallel β-strands (β3 and β4) and helices (α2, α3, α4 and α5). *Yersinia* CMP kinase can be described as a member of the class of long-NMP kinases owing to the insert found in the NMP-binding domain, which is not present in short variants: in this structure, the insert comprises the anti-parallel β-strands, a loop region that connects β4 to β4, and α5. The lid domain is composed of helices α7 and α8. *Yersinia* CMP kinase and *E. coli* CMP kinase (PDB code 1CKE) superimpose on each other with a root mean square deviation (r.m.s.d.) of 210 Cα atoms of 0.91 Å. The proteins share 84 per cent identity and 93 per cent similarity.

**Figure 4.**
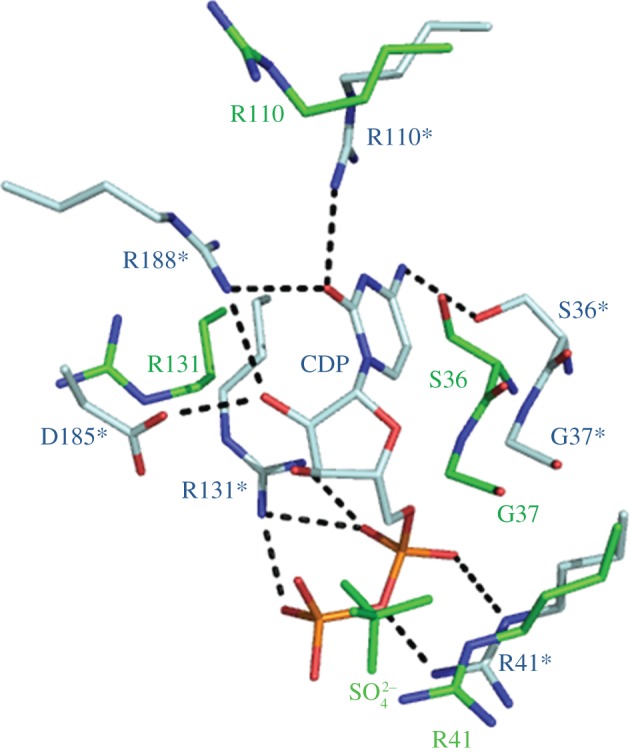
Active site of CMP kinase. Green sticks: positioning of the sulphate ion at the CMP kinase active site and a number of surrounding residues shown. Blue sticks: model of CDP-bound CMP kinase is shown and residue labels are denoted with an asterisk. Predicted hydrogen bonds are shown as black dashes. This figure was prepared using PyMOL.

The ligand-binding site is formed by a pocket on the NMP-binding domain. In related structures, CMP and CDP bind such that the cytosine base of the ligand sits at the C-terminal end of the β5 strand, and the phosphate moiety is positioned next to the α2–β2 boundary [8,9]. One sulphate ion is bound at this site (figure 4). Residues Glu180 to Ala191 are disordered in this structure. This is also the case for *E. coli* CMP kinase (PDB code 1CKE), where residues 180–192 are disordered in the absence of ligand.

Within the structure, an anion hole is formed by a glycine-rich loop that forms part of the mononucleotide-binding motif. This forms the ATP-binding site, and in the *Yersinia* CMP kinase, a sulphate ion is bound. The sulphate ion occupies the same position as that of previously published bacterial CMP kinase structures that have been crystallized in the absence of ATP [2,33].

### 4.3. The CMP kinase: CDP model

**Figure 5.**
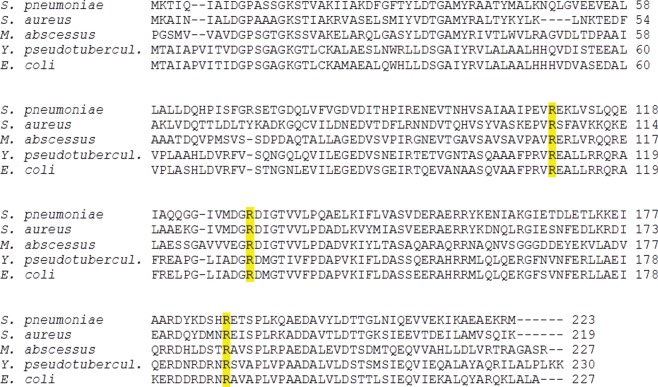
Sequence alignment between *Streptococcus pneumoniae*, *Staphylococcus aureus*, *Mycobacterium abscessus*, *Yersinia pseudotuberculosis* and *E. coli* CMP kinase. Residues that were mutated in the *Yersinia* protein for this study are highlighted. Alignment was performed using the ClustalW sequence alignment program [34].

A model of *Yersinia* CMP kinase with CDP bound was created based on the sequence alignment between *Yersinia* CMP kinase and *E. coli* CMP kinase with CDP bound (PDB code 2CMK). The proteins share 84 per cent identity and 94 per cent similarity, with all ligand-interacting residues being conserved (figure 5). We have compared the structure of *Yersinia* CMP kinase in the absence of ligand with the model of CDP-bound *Yersinia* CMP kinase in order to propose potential binding-induced changes for *Yersinia* CMP kinase.

The NMP-binding domain undergoes movement upon substrate binding, most notably in the anti-parallel β-strands β3 and β4. The α5-helix moves towards the binding site, giving a more closed conformation. The homology model contains a section from residues E180 to A191, which is not seen in the apo-enzyme. In the homology model, this section is mainly α-helical and is an extension of the α8 helix. Residues Arg188 and Asp185 from this section interact with CDP in the homology model. This is also the case in *E. coli*, and Briozzo *et al.* [2] suggested that this is why the region becomes ordered upon binding. The most notable side-chain rearrangements are those of Arg131, Arg110 and Arg41. Arg131 and Arg41 side chains make hydrogen bonds with the phosphate moieties of the ligand. Arg110 side chain forms hydrogen bonds with the cytosine base of CDP, as does the nearby Asp132 side chain. Residues Ser36 to Ala38 at the N-terminal end of α2 helix are predicted to move away from the ligand-binding site, and there is a predicted hydrogen bond between the Ser36 side chain and the cytosine base. Adjacent to this section, Tyr40 moves such that a stacking interaction can be made with the cytosine base of CDP. The phosphate moiety of the ligand is next to the α2–β2 boundary and Ser14 is hydrogen bonded to CDP.

### 4.4. Activity of *Yersinia* CMP kinase

The activity of the CMP kinase was confirmed in an *in vitro* assay. At a constant concentration of 1 mM ATP, the *K*_m_ and *k*_cat_ values for CMP were 0.028 mM and 91.9 s^−1^, respectively, and at a constant concentration of 0.1 mM CMP, the *K*_m_ for ATP was determined to be 0.04 mM with a *k*_cat_ of 74.3 s^−1^. On comparison of the activity data available in the literature (table 2), the *K*_m_^CMP^ of the *Yersinia* CMP kinase is similar to that from *E. coli* and *B. subtilis*. The *K*_m_^CMP^ for the *M. tuberculosis* protein, however, is approximately threefold higher than that from the other bacteria, with a *k*_cat_ twofold lower than the *Yersinia* and *E. coli* proteins. Both the *Yersinia* and *E. coli* CMP kinases are inhibited by CMP over 0.2 mM; however, the *B. subtilis* protein is only slightly inhibited by concentrations over 2 mM [37]. The *Yersinia* protein *K*_m_^ATP^ is again similar to that of the *E. coli* protein, but the *B. subtilis* protein *K*_m_^ATP^ is threefold higher.

**Table 2.** Summary of the kinetic data for the *Yersinia* CMP kinase and other bacterial CMP kinases available in the literature.

		CMP		ATP	
	reference	*K*_m_ (mM)	*k*_cat_ (s^−1^)	*K*_m_ (mM)	*k*_cat_ (s^−1^)
*Y. pseudotuberculosis*	this study	0.028	91.9	0.04	74.3
*E. coli*	[5,35]	0.035	103	0.038	—
*M. tuberculosis*	[36]	0.12	52	—	—
*B. subtilis*	[37]	0.04	—	0.12	—

On the basis of structural analysis, site-directed mutants were generated in residues predicted to play a key role in the active site. In order to assess whether the mutants were correctly folded, CD experiments were performed on wild-type CMP kinase and each of the mutants Arg131Ala, Arg110Ala and Arg188Ala. The CD spectra for the proteins were highly similar, indicating that the mutants were correctly folded. When the mutated proteins were assayed at a concentration of 10 µM with a constant concentration of 1 mM ATP and concentrations of CMP up to 5 mM, no activity was seen, showing them to be essential for CMP binding in the active site.

## 5. Discussion

The need for new antibiotics has become pressing in light of the emergence of antibiotic-resistant strains of human pathogens. *Y. pestis*, the causative agent of plague, is a public health threat in some parts of the world and multiply antibiotic-resistant strains have been reported [17], and thus there is a need for effective new antibiotics to treat this notorious disease, which has been responsible for millions of deaths in history. Unfortunately, the need for new antibiotics is even more pressing for *Y. pestis* as the organism has long been of concern in biodefence. Previously, we had reported the identification of *cmk* as a potentially interesting and essential gene that we could target for development of novel antibiotics [13]. In this study, by modifying the approach to mutating the target gene, we were able to produce a mutant of *Y. pseudotuberculosis* in which *cmk* was inactivated. The homologous DNA sequences in the oligonucleotide primers were the same, the only difference being that in the successful approach the kanamycin-resistance cassette was driven by the *cmk* promoter rather than by the kanamycin cassette promoter. This may explain conflicting reports of essentiality in some organisms as the mutant appears highly sensitive to which approach is used during production.

Similar to *E. coli* [8], CMP kinase is required for normal growth of *Y. pseudotuberculosis* at 28°C. Owing to issues of virulence plasmid instability at 37°C, we did not evaluate the growth defect at 37°C, but it would be assumed that there would be a growth defect at this temperature also. As predicted by our bioinformatic analysis [13], the *Y. pseudotuberculosis Δcmk* mutant was significantly attenuated *in vivo*, thus confirming it as a potential target for novel antimicrobials for the pathogenic *Yersinia*. This is the first report of *in vivo* characterization of a *Δcmk* mutant, as in the few instances where a mutant has been generated the published studies have primarily focused on the impact on bacterial physiology [8,9]. The fundamental physiological function of CMP kinase is the phosphorylation of CMP produced during turnover of nucleic acids to produce the di- and tri-phosphates, and inactivation of *cmk* results in increased internal levels of CMP and excretion of uracil and cytidine [8,9]. As CTP is required for DNA synthesis, the loss of CMP kinase must be compensated for in *Y. pseudotuberculosis* in a similar way to *S. enterica*, where *de novo* synthesis of CTP by the CTP synthetase encoded by *pyrG* maintains a supply of CTP [8,9]. In addition to being a precursor for RNA and DNA synthesis, CTP and dCTP are also involved in phospholipid biosynthesis. Therefore, there may also be effects of inactivation on membrane synthesis, which could also affect growth rate and may contribute to attenuation. It is well documented that even apparently minor changes in membrane composition, such as those induced by mutation of the PhoPQ regulon [38], for example, can have a large impact on virulence.

Although the *Yersinia* are closely related to *E. coli*, both being members of the Enterobacteriaceae, the NMP kinases may have different biochemical and physico-chemical properties. For example, despite a high degree of amino acid identity and the conservation of nucleotide binding and catalytic amino acids, thymidylate kinase from *Y. pestis* and *E. coli* exhibited significant differences in thermodynamic properties and phosphorylation activity [39]. However, such a difference was not observed for CMP kinase. The reported *K*_m_ for *E. coli* CMP kinase is 0.038 for ATP as a phosphoryl donor and 0.035 for CMP as the phosphate acceptor [5]. This is very similar to *K*_m_ values for the *Y. pseudotuberclosis* protein of 0.04 and 0.028 mM, respectively.

The structure of *Yersinia* CMP kinase reveals a globular protein consisting of three domains. This is the same overall fold that is observed in related NMP kinases. *Yersinia* CMP kinase contains an insert in the NMP-binding domain, characteristic of the long-NMP kinases. In the absence of a structure in which ligand is bound, homology modelling has predicted a number of changes that take place upon ligand binding and a number protein–ligand interactions have been predicted. These interactions are based on the high identity between *Yersinia* and *E. coli* CMP kinase. The importance of three key arginine residues at positions 131, 110 and 188 in particular has been confirmed through site-directed mutagenesis, as substitution of these key amino acids showed them to be essential for CMP binding in the active site. Such detailed insights into the stereochemistry of substrate binding by CMP kinase will underpin future structure-based efforts to develop novel inhibitors targeting the CMP-binding site.

## 6. Acknowledgements

This work was supported by the Ministry of Defence and a Wellcome Trust (UK) equipment grant no. 088464 to K.R.A. We thank the scientists at station IO3 of the Diamond Light Source (Didcot, UK) and Dr Nethaji Thiyagarajan for support during X-ray data collection, and Rebecca Driesener at the University of Southampton for help with enzyme kinetic data analysis. We also thank Marc Baylis for technical support. The atomic coordinates and structure factors (code 4E22) have been deposited in the Protein Data Bank, Research Collaboratory for Structural Bioinformatics, Rutgers University, New Brunswick, NJ (www.rcsb.org).
